# A Report on Epidemics and Endemics

**Published:** 1861-01

**Authors:** O. C. Gibbs

**Affiliations:** Frewsburg, New York


Art. II.—*A Report on Epidemics and Endemics.* Read before the Medical Society of Southwestern New York, at its several Sessions during 1848. By O. C. Gibbs, M.D., of Frewsburg, New York.

Man, formed from the dust of the earth, has ever shown a proclivity to return to his original element; hence, from the earliest records to the present time, disease, and pain, and death have been the common heritage of the race. The ways in which disease gains a victory over life, and death claims its victims, are multiform and various. There are affections which occur independently of all known external influences; there are others depending upon known and common causes; to both of which classes man is exposed in all countries, climates, and conditions of society. There are diseases which owe their existence to a combination of causes, perhaps at present beyond the jurisdiction of human understanding, which attack thousands of individuals at the same time, leaving sorrow, and death, and desolation in their train; there are other diseases that occur only in certain localities, or under a certain circumscribed combination of causes; and, although less direful in their results, they are of more frequent occurrence, and, consequently, none the less worthy of attentive consideration. The former of this latter division are denominated Epidemic, the latter Endemic diseases.

Some epidemic diseases have confined their ravages to the Old World altogether, never having visited the American Continent; others, though they may have originated in the jungles of India, have made fearful and repeated visitations to this country, and, at last, become naturalized on the Mexican Gulf and the Caribbean sea-coast, and in the hot and humid delta of the great Mississippi; all, with few exceptions, confine their ravages to the larger towns, or to low, damp, and filthy localities: hence we, mostly laboring in rural districts, and on the airy hills of Chautauque, 1300 feet above the level of the sea,* have really but little to fear from yellow fever, cholera, or the plague. Yet, notwithstanding our comparative exemption, I trust that we are not indifferent to aught that has connection with medical science by the close affiliation of disease and death. Few subjects have attracted greater attention, and none have excited more terror and dismay, than those terrible epidemic visitations that have occasionally decimated a nation, a continent, or a world.

In discharging the duties which are assigned to me on this occasion, I would, perhaps, act the wiser part in confining my remarks to diseases which are endemic in this locality. I propose, however, a wider field of inquiry; and should I allude to diseases which some of us perhaps have never seen, and never will, I shall do so, not to impart information in regard to them, but simply to illustrate the central idea of this paper, which is, that *all epidemic and endemic diseases have a particular climate and geographical position, and atmospheric and terrene relations.* It was, probably, Baron Humboldt who first called attention to the isothermal relations of plants. The diseases under consideration are no less bounded by certain isothermal lines, and have relation to geographico-meteorological causes. They have a certain natural geographical order and distribution, regulated by the conditions of the soil as to drainage, the climate as to temperature, the air as to humidity, density, and electrical condition.

The earliest preserved records of the human race bring us tidings of the fearful ravages of epidemics and pestilential diseases. Sacred history makes allusion to the plague "that wasteth at noonday." Homer speaks of its ravages in the Grecian army while encamped before

* The height of Chautauque Lake.

Troy; and Sophocles gives a terrible picture of the desolation of Thebes. But these records, handed down to us through the mist of ages, are too meagre in facts and detail to serve any other than historic purpose.

The Plague.—More than 400 years before the Christian era, in the days of the Peloponesian war, the plague ravaged the populous City of Athens and the surrounding country, adding its gloomy horrors to the devastations of fire and sword of an invading army, carrying death wherever it went, and leaving little behind, save utter despair and desolation. A thousand years later, or in the sixth century of the Christian era, it ravaged the Roman Empire. For a time, it is said ten thousand persons died daily at Constantinople, and many cities of the East were nearly or quite depopulated. Gibbon estimates the number who perished in this epidemic, which lasted for several years, at between one and two millions! This unsparing devastator of the Levant made its first appearance, it is supposed, in Europe, in the year 1348. In Italy, France, and England the mortality was terrible. It has been supposed that three-fourths of the inhabitants of this region of country perished. Florence, in Italy, was nearly depopulated. Still a thousand years later, or in 1665, the plague visited London, causing a terrible mortality, and inflicting physical suffering, mental despair, moral turpitude, and fiendish disregard of the humane dictations of all the higher impulses of man's nature, of which the imagination can at present form but a faint conception. In this plague-smitten city, it has been estimated that 3000 perished in one night, and the whole mortality has been set down at 100,000. Nearly a hundred years later, or in 1720, this epidemic raged at Marseilles, on the Mediterranean sea-board of France, with a severity and mortality perhaps even greater than in London in 1665. But there is one fact that in some measure relieves this gloomy picture of death and desolation; the sick were not abandoned to their fate; medical aid, hospital regulations and religious consolation, so far as was possible under the circumstances, were secured to all sufferers. In 1816 this epidemic was very destructive in the Ottoman Empire; and as late as 1841, it raged in Syria and Erzeroum with great violence.

Mention has been made of only a few of the many visitations of this disease, but sufficient, I trust, to answer my present purpose in alluding to its geographical, atmospheric, and climatic relations. It will be seen that the endemic seat of the plague is on the eastern shores of the Mediterranean; there it has continued to rage, at uncertain intervals, from the earliest records to the present time. From thence it has spread southward along the valley of the Nile and the Red Sea, westward along the valley of the Mediterranean, and eastward along the low lands of the Black and Caspian Seas and the Persian Gulf; mostly between the parallel of latitude 25° and 45° north. "It seldom extends to the southward beyond Siout, in the valley of the Nile, or Jiddah, on the Red Sea. In Asia it prevails chiefly on the coasts of Syria, and a portion of the shores of Asia Minor, where it sometimes ascends the river valleys. In Europe it is endemic only on a part of the eastern coast of Turkey. It has never yet appeared in the southern hemisphere, nor in America."*

It is true, when the plague visited Paris and London, and even farther north, it passed beyond the geographical limits above specified; but it is highly probable that some peculiarity of season gave rise to a climatic change, analogous to that which is natural to the shores of the Mediterranean, and, consequently, not disproving the isothermal relations of the disease. It will be observed that the appearance of the plague is limited to the lower portions of the earth's surface; appearing mostly near the sea-shore, and at but a slight elevation above the surface of the ocean. Johnston, in the work above referred to, says: "When it is ravaging the lower quarters of Constantinople, the inhabitants of the higher portions of the seven hills, on which the city is built, often escape altogether; and Brayer mentions a village, situated on Mount Alem Dagh, at an elevation of about 1600 feet above the sea, where it was never known to appear, and which was resorted to as a place of refuge for the citizens; and there is a place in Malta, hitherto inaccessible to the disease, and on this account called *Safi* (pure.) It is recorded by the French physicians, that during their occupation of Cairo, the plague never reached the citadel of that city; and Clot Bey states that it, as well as the village of Loumeldik, situated at a considerable elevation, was spared during the epidemic of 1835."

Humidity in the atmosphere, and an argillaceous soil or subsoil, are other conditions in the production of the plague, to which I shall have occasion again to refer more in detail, when speaking of other epidemics. He who will take the trouble to examine the history of the various manifestations of this epidemic will be surprised to learn how uniformly it confines its ravages to cities situated on low, marshy sites, on the banks of rivers, or near the sea-shore—elevated, dry, airy, and well-drained localities are uniformly exempt from its ravages.

Having instanced a high degree of temperature, humidity of the atmosphere, and dampness of soil, as causes favorable to the production of this epidemic, I shall mention but one other, and that, perhaps, is more potent than any one to which allusion has been made, although mainly dependent upon their combined action for its existence. I mean atmospheric poisoning from the decomposing exhalations incident to an

* See A. K. Johnston on the Geographical Relations of Health and Disease.

ill-ventilated, ill-drained, and over-crowded city, as well as from the noxious gases arising from animal and vegetable decomposition. The records concerning the terrible visitation of this disease at Athens, more than two thousand years ago, are in full confirmation of these statements. It is said that the winter preceding this epidemic was unusually rainy, and that the succeeding summer was unusually hot, untempered by the usual Etesian winds. It should be remembered that Athens was situated in a plain, surrounded by mountains on all sides save one, in which direction it was open to the spray and dampness of the sea, and, at that time, excepting its magnificent public buildings, consisted mostly of a collection of squalid huts and filthy streets. The terrified people from the country rushed to the city for protection from the fire and sword of the Peloponesians; and an able writer says: "Every vacant piece of ground, not excepting the sacred inclosures of the temples, was whitened with the tents of the fugitives. The towers were occupied, and the increasing throng filled up with their temporary huts the space between the long walls, and even placed casks in the recesses to accommodate those who could find no other shelter."*

Other results could not have been expected, than that disease, under these circumstances, would work unprecedented ravages among the squalid people of an over-crowded city; hemmed in, as were the residents, by the surrounding hills, from the invigorating breezes and their healthful influences, subject to the burning heat of a tropical, summer sun, and depressed and poisoned by the humid exhalations from a saturated soil, and the effluvia incident to an over-crowded and filthy city. The same combination of causative circumstances accompanied the appearance of the plague in London, nearly two centuries ago. The army of Cromwell had just been disbanded, and the men of whom it was composed returned to London to participate in the vice and dissipation of that then dissolute city. The population of London at that time has been estimated at half a million. The city was mostly composed of wooden buildings, the streets were narrow, undrained, never cleaned, and constantly reeking with unspeakable abominations.

Sydenham says the preceding winter was extremely severe and protracted, the opening of spring was late but sudden, and the heat of summer increased steadily to an almost unbearable culmination as late as

* See *Harper's Magazine*, vol. xiii. p. 63. I wish here to acknowledge my indebtedness to a series of articles on *Great Epidemics*, published in this magazine, from which I have drawn many of the foregoing facts. Should I, in the continuance of this report, find in that series any facts that may serve my present purpose, I shall avail myself of them without, perhaps, again acknowledging my obligation.

September. Under these circumstances, the plague seemed almost like a special providence, designed to awaken the stupid understanding of man to the fact that filthy dens and haunts of vice are ever the hotbeds of disease and death. These are by no means isolated cases. Constantinople, Florence, Marseilles, Syria, etc., each presented a combination of causes of disease in no way inferior to those which proved so destructive in Athens and London.

These facts surely indicate that the plague is not a meaningless curse, sent only to depopulate a world, but rather is a visitation, permitted by Providence, as a necessary consequence of a criminal disregard of those conditions essential to human health and welfare. They indicate plainly that man is not always, of necessity, to suffer and to want; but that, when he fulfills the demands of a high and Christian civilization, the devastations of the plague will be known only in history.

YELLOW FEVER.—The next epidemic disease which I propose to discuss, is yellow fever; and, like the plague, it hides its origin in the dimness of antiquity, but, unlike that epidemic, has not confined its ravages to the Old World. It is invested with peculiar interest to us, from the fact that it has become naturalized in the sunnier portions of the United States, and makes almost annual excursions northward. In fact, it has not been long since we were gazing sadly upon, and sympathizing helplessly with, two afflicted cities not far to the southward—Norfolk and Portsmouth—and the shadows of grief and mourning occasioned by the ruthless ravages of the pestilence yet hang heavily on many hearts.

Yellow fever has repeatedly ravaged cities on both continents and both hemispheres, yet its northern isothermal boundary stops short of that which circumscribes the plague.

It cannot be expected that I will here allude to the various epidemic visitations of this disease, appearing in this country and elsewhere, nor instance the thousands that have been swept away by its tornado-like visitations at New Orleans, Norfolk, Philadelphia, and elsewhere in the United States; at Vera Cruz, in Mexico; Havana, in Cuba; Georgetown, in British Guiana; Rio Janeiro, in Brazil; or Barcelona, in Spain. Limited as is my time and space, I propose to deal less with historic record than with the geographical relations of the disease under consideration—its atmospheric and terrene causative phenomena.

At the close of the seventeenth and the beginning of the eighteenth centuries this fever was, perhaps, more extensively prevalent as an epidemic, and more fatal in its attacks, than at any former time of which we have record. In 1793, in Philadelphia alone, more than four thousand died of the disease in a few weeks, and at one time the vomit-pestilence swept its victims into eternity at the fearful mortality of more than a hundred a day. Nearly all business was suspended save caring for the sick and burying the dead; the demand for coffins was more insatiable than the demand for bread; and the roads to the cemeteries were the only ones not deserted. In 1795 nearly four hundred died from the disease in Baltimore; and three years later, nearly six hundred. In 1798 Philadelphia and New York were terribly devastated; in the former city nearly three thousand perished, and in the latter more than two thousand. In the year 1800 the fever prevailed extensively in New York, Baltimore, Providence, Charleston, and Norfolk, and raged with still more terrible severity in cities on and near the Mediterranean coast in Spain. In Seville alone, it is estimated that more than twenty thousand persons perished. In 1804 Spain was again the seat of its most ruthless devastations, in which kingdom, in that year, it is supposed that it destroyed nearly sixty thousand lives. From Malaga alone twelve thousand went to swell the mortality records, and the city was rendered desolate. Business, trade, pleasure, religious observances, everything was suspended, save death, which went ruthlessly on gathering victims from every house which desertion had not rendered tenantless. The sick groaned alone, with no ear to hear, nor heart to sympathize, and often drew their last breath untended and unwatched by friend or kindred. Parentless children went weeping, and ownerless dogs howling through the streets, adding the evidences of woe to desolation.

After 1805 the yellow fever ceased to excite any alarm as an epidemic, until 1819, in which and the following year it fully made up in general prevalence and fatality of attack what it had lost by a few years of quiescence. New Orleans, Mobile, Natchez, Charleston, Baltimore, Philadelphia, New York, and several other towns of less note, were almost simultaneously attacked and suffered terribly. During the same years it raged with great fatality in the West India Islands, at Vera Cruz, in Mexico, and in various cities in Spain. At Cadiz alone, in 1819, five thousand perished from the disease.

For nearly thirty years the fever again ceased to rage as an epidemic. In 1850 it commenced anew its ravages; Rio Janeiro, in Brazil, was swept as with a besom of destruction. In a few weeks, in that city, and on board the becalmed vessels in its port, ten thousand hearts ceased to beat, and ten thousand human forms found a hurried sepulture. For the next five years the fever continued to prevail, progressing northward. In 1852 the West India Islanders, and the dwellers upon the shores of the Caribbean Sea and the Mexican Gulf, severely felt its ravages. In 1853 it revisited its old haunts in the United States; and its fatality can be judged of when it is remembered that more than eight thousand persons perished in New Orleans. The appearance of the disease at Norfolk and Portsmouth, and the more than four thousand deaths occasioned by it, in 1855, is too fresh in our minds to need further remembrancing.

The above brief allusion to some of the principal manifestations of this affection, I trust will prove sufficient to indicate its geographical relations, and fix in some measure its terrene causative dependencies. You will observe one point of difference in the geographical distribution of yellow fever and the plague: the latter confines its ravages principally to a certain zone in the northern equatorial half of the Eastern Continent; the former, although it has latitudinal limits, finds its victims in both continents and both hemispheres. You will also see one point of resemblance; for while the plague does its deadliest work upon the low, damp, and hot coasts of the Mediterranean and the Levantine seas, the yellow fever commits its ravages in similar situations upon the hot coasts of the Atlantic and the steaming deltas of its many rivers. You will doubtless observe, also, that while yellow fever visits Rio Janeiro, Havana, New Orleans, Philadelphia, and other cities, it does not visit Oriental cities similarly situated, as, for instance, Athens, Florence, and Constantinople. It will doubtless strike you as even more singular that, while yellow fever desolated Malaga and Barcelona, in Spain, it was the plague that committed such terrible ravages at Marseilles and Avignon, in France. The reason for this geographical arrangement of the two diseases it would certainly be interesting to know, and I shall have occasion to refer to the subject again before concluding this paper.

Yellow fever is emphatically a disease of hot weather and warm climates, occurring mostly between the 25th parallel of south latitude and the 35th north. It is true, it has passed the 40th degree north in visiting Barcelona, in Spain, and New York and Boston, in the United States; but such incursions northward have only been made when favored by a tropical identity of climate. It should be remembered, however, that it is not degrees of latitude that bound the disease, but that it is circumscribed by isothermal lines, represented by an average temperature of about 80° F.

Dr. Barton, of New Orleans, says that a temperature above 90° is too high for the production of yellow fever. This statement probably needs confirmation; for in 1854, on one occasion, it is said the thermometer, in Charleston, stood at 93° at midnight, and that for four months the average temperature was 80°; yet the fever was never more fatal than in that city at that time; even negroes who were born there were not exempt from its fatal ravages. It will be remembered that Charleston is between the 32d and 33d degree of north latitude, and it is highly probable that at Georgetown, in British Guiana, which is in about the 6th degree of latitude, and at Vera Cruz, which is below the 20th degree, the temperature at times ranges above 90° F. I regret that I need statistics of the average temperature of the two places last named; but I am certain that the fever has prevailed repeatedly in both, and always during, or immediately subsequent to the hottest season of the year. Heat, however, is not a direct cause of yellow fever; it acts secondarily through its influence upon other atmospheric and terrene conditions.

In speaking of the plague, I said that any person who would take the trouble to examine the history of the various manifestations of that epidemic, would be surprised to see how uniformly it confined its ravages to cities situated on low, marshy sites, on the banks of rivers, or near the sea-shore. I now propose to show that, as to site, the predilections of yellow fever are the same, and that, to all appearances, the terrene causative phenomena are identical in the two diseases.

Commencing then with Rio Janeiro, it is unnecessary to remark that it is situated nearly in latitude 23 degrees south, and on the western shore of a bay opening into the Atlantic. The site of the city is a marshy plain. Half a mile back from the shore the elevation is only five feet above the level of the sea. The back ground is composed of precipitous mountains, varying in height from 1500 to 3000 feet. The soil is an alluvia, composed principally of clay and vegetable mould. "The granite hills around the city, as well as the expanded surface of the bay, constitute reflectors of the calorific rays which augment the temperature and humidity, which are not abated much by the winds, because they are interrupted by the disposition of the mountains."*

The streets are narrow and imperfectly paved, and, in consequence of the levelness and lowness of site, all the filth incident to a maritime city either reeks in the full blaze of the summer's sun, or sinks just below the surface of the soil, to stagnate, and putrefy, and poison the air with its noxious exhalations. Added to all this, its geographical position makes it a commercial centre of trade with the different nations of Europe and Asia, also with Oceanica; consequently the ships of nearly all nations congregate in its port.

In almost exactly the same degree of latitude north that Rio Janeiro is south, stands Havana, in Cuba, to which and to Vera Cruz, in Mexico, which is about four degrees farther south, I invite a moment's attention, as yellow fever has prevailed endemically in these maritime cities ever since it excited much attention as a distinct disease. Havana stands on a plain facing the sea to the eastward, and, like Rio Janeiro, is surrounded in all other directions by high hills, which obstruct the winds and concentrate the heat. "In one corner of the plain and near the harbor is a

* Medical Examiner, vol. xi p. 645.

swamp, the exhalations from which are wafted over the city and shipping."* The Gulf Stream brings to the coast and the waves throw upon the shore an immense quantity of sea-weed, the decomposition of which, under the action of an almost tropical sun, adds poisonous exhalations to an atmosphere already impure.

Dr. Dupierris, of Havana, gives a sad picture of the hygienic condition of this city, even as late as 1857. I have space for only a short quotation. "The public cemetery is here a nuisance. The soil is sandy and only two or three feet deep, and rests on a hard rock. The emanations from the decomposition of the bodies deposited here are sensibly felt." "The streets are for the most part narrow, badly laid out, unpaved, with the surface broken into irregularities, and obstructed with impurities. In other places one is disgusted with the sight of filthy ponds or stagnant pools, apparently to receive rain water, the refuse and offal from houses, and a heterogeneous mixture of all kinds of abominations, among which are included dead animals and the like."†

Vera Cruz is the largest city on the Western Gulf coast, and, like the two cities above mentioned, is situated on a plain facing the sea and surrounded by hills. In the rear of the city, and in front of the hills, are large swamps, which in the hot season always load the air with miasm, and whose waters filtrate to the cellars and wells of the city.

Norman, in 1845, said of the city: "The outside looks solitary and miserable enough. The ruins of deserted dwelling-houses, dilapidated public edifices, neglected agriculture, and streets once populous and busy now still and overgrown with weeds, give an air of melancholy to the scene, which it is absolutely distressing to look upon."‡

Passing south to the sixth degree of north latitude, I would for a moment direct your attention to Georgetown, situated at the mouth of the Demerara River. This is a large town, situated on an alluvial plain, below the level of the sea, from which it is protected by embankments. The buildings are of wood and are raised from the ground on pillars. To the east and north of the town, on the front lands of the coast, are many thousand acres of jungle. Dr. Blair, surgeon-general of the English army division stationed at British Guiana, says: "To the north, during the time of the epidemic, there also existed a naked marsh of about two hundred and fifty acres. During each high spring tide the sea covered the surface of the marsh. The soil was composed of the usual constituents of our foreshores, viz., clay, caddy, and drift mud. The marsh was tufted

* Drake's Diseases of North America, vol. i. p. 47.

† North American Medico-Chirurgical Review, vol. i. p. 873.

‡ Norman's Notes of Travel, p. 96.

with a coarse grass, under whose half-withered leaves myriads of insects were sheltered; innumerable crabs burrowed throughout; fragments of drift-wood, bones, dead spawn, dried molusca, and small fish, left by the retreat of the tide, were scattered profusely over the surface."*

A town situated on such a site, with such surroundings, under the burning influence of a tropical sun, supplied with no water save what infiltrates from the marshes, could only be expected to be unhealthy; and no one will be surprised to learn that in 1837 *sixty-nine* per cent. of the English forces quartered there perished with yellow fever.

Passing northward to the thirtieth degree of latitude, I would call your attention to New Orleans, which is situated on a low, alluvial plain, on the left bank of the Mississippi River, below the level of that river at high water, and only ten and a half feet above the level of the sea. New Orleans is mostly surrounded by lakes, at distances varying from four to thirty miles; thus directly north and nearest is Lake Pontchartrain; east is Lake Borgne; and on the south is Lake Ouacha and other smaller lakes; to the west they are absent, but in that direction the broad bosom of the river stretches away meanderingly for a distance of seventy miles. "Even this presents an inadequate idea of the extent of watery surface, for in every direction from the city, unless when we travel on the 'coast' or river bank, we encounter cypress swamps, terminating either at the shores of a lake or in grassy savannas too wet to be traveled over. Before levees were raised upon the banks, the whole region was annually overflowed; but during nine months a strip on each side, varying from a few yards to a mile in width, was dry on the surface, yet abounded in water underneath."† Even now the ground upon which the city stands is so damp that water can be obtained anywhere at the depth of a few feet, which water either infiltrates from the river or the stagnant marshes. It must be evident that, during the hot season of the year, the swamps in the rear of the city must fill the humid air with their poisonous exhalations. It is not from the rear of the city only that the seeds of disease and death emanate; for the streets adjacent to the river, for three miles, are compactly built up with warehouses, market houses, drinking houses, oyster houses, sugar wharves, cotton presses, etc., from which issues a vast quantity of filth and organic recrements, which foul stratum reeks in the burning sun of the thirtieth degree of latitude, and sends forth noxious exhalations to commingle with those from the swamps, the filthy streets, and the well-filled cemeteries of the city.

---

* Account of the last Yellow Fever Epidemic of British Guiana, by Daniel Blair, M.D., p. 5.

† Drake, on the Diseases of the Mississippi Valley, vol. i. p. 98.

It may be supposed that the beautiful and usually healthful City of Philadelphia, which has repeatedly been desolated by the yellow fever, would present an exception to those atmospheric, geographical, and terrene sources of disease, which have been found to obtain in the other cities to which special allusion has been made. But an examination will show that it does, or at least has presented in abundance the same causative elements of the fever which have been found to exist elsewhere. Philadelphia is situated upon a low and level tract between the Delaware and Schuylkill Rivers. The soil upon which the city stands is argillaceous to some depth, though underlaid by a tertiary formation of sand and gravel. Various marine deposits show that the site of the city was once covered by the sea, and so retentive of moisture is the soil, that water is usually found at the depth of ten or twelve feet. The wharves or quays extend for several miles along the margin of the Delaware, and are built of square casements of logs, filled up with earth and stones. These square casements, projecting into the river, include open spaces or docks between them, the water in which is cut off from the current of the river. It is into these spaces that the gutters and sewers empty the filth of the city; all the debris accumulating in the warehouses, market houses, drinking houses, and upon the wharves, finds there a lodgment, which nauseous elements of putrefaction, conjoined with the filth brought there by high tide, remain, and in the heat of the summer's sun decompose and fill the air with offensive and poisonous effluvia.* Back of the wharves, and running from one extremity of the city to the other, is Water Street, which has always played an important part in yellow fever epidemics. "The houses on the western side of the street are built against the elevated ground, which was once the back of the river, and hence the lower story at the back part is constantly damp, and even wet at times, by the percolation of moisture from the earth, against which the lower part of the wall rests. There is, of course, no yard room, and the privies are placed in the cellars. On the other or eastern side of the street, nearest to the river, the houses back on the stores or warehouses, which are built along the wharves facing the river; and here again is a want of common space for yard, so as to allow of ventilation, or the construction even of privies. The street itself is narrow, and lined on both sides by high houses, once occupied by the better and some of the wealthier classes; but for a long time past they have been either converted into warehouses and petty shops, or are tenanted by lately-arrived emigrants, poverty-stricken residents, and sailors."† The neck of land below the city is low, flat, and

* See a fuller description, of which this is but an imperfect and condensed one, in La Roche's valuable work on Yellow Fever.

† See Medical Examiner, vol. xi. p. 596.

marshy, and was formerly an unreclaimed swamp whose waters stagnated in the summer's heat. There were, in earlier times, other elements of disease at work which are now nearly or quite eradicated. Space will not permit their full enumeration; suffice it to say, that the city plot was once partially traversed by small streams, the principal of which were Dock Creek and Pegg's Run. These streams received the offals of many slaughter-houses, tan-yards, glue, starch, dressed skin, soap manufactories, and stables adjoining, as well as the contents of culverts, privies, and the gutters of populous streets and filthy alleys. These miry streams, in the hot weather, were often destitute of water; consequently the accumulating filth putrefied in the sun, and sent up highly offensive and noxious exhalations.*

Perhaps I have dwelt longer upon the geographical relations and terrene dependencies of yellow fever than seems necessary, but I am loth to quit the subject. So far it has been shown that those cities which have felt the ravages of the fever most have been low, damp, and hot, in the vicinity of stagnant marshes, and containing without their very precincts all the elements of disease, which arise from defective sanitary regulations.

He who will take the trouble to examine for himself will find that protracted high temperature approximating the elements to a tropical one, accumulations of vegetable and animal matter, acted on by heat and moisture, defective sewerage, filthy streets, confined, damp, and badly ventilated habitations, characterize all places that, at different times, have been ravaged by the disease.

I will mention but one other terrene condition having relation to yellow fever as a probable favoring cause; I have reference to the character of the water used by the citizens in the affected localities. Dr. Barton truly says, "town water is town air," and it is highly probable that impurities, entering the stomach with the former, are not less deleterious than when they enter the lungs with the latter. It cannot be doubted that water sometimes holds in suspension or solution matters positively injurious to health. Thus, when it percolates from marshes, it may carry with it putrescent organic matter prejudicial to health, also when it infiltrates from graveyards and the vaults of privies, it may contain the poisonous elements of animal decomposition. It is said of the earlier visitations of yellow fever in Philadelphia, that "at that time no water works had been erected, and the inhabitants were compelled to use wells, which, being shallow, were tainted with all the impurities which can filter through the soil of a city." Devèze says: "The drainings of the graveyards found their way into some of these wells, and the water from them became

* La Roche on Yellow Fever.

putrid within twelve hours from the time it was drawn."* The water used in most of the cities in which yellow fever has prevailed has been rendered impure from a combination of both these causes. Bordered by marshes, the sites of the cities low, undrained, and, in most instances, unsupplied with water-works, the wells from which the necessary supply of water has been drawn have been replenished by percolations from swamps, vaults of privies, tan-yards, stables, and the many other sources of impurity which characterize all large cities. With the exception of water, it will be observed that terrene conditions of the disease act meteorologically, or through the medium of the atmosphere. The water actually used is really the only exception, for the unused water, when exposed, will, as well as animal and vegetable putrescence, exhale its noxious properties into the surrounding atmosphere. Thus "the putrid water of the Cove in Baltimore, with its mantle of green slime broken by bubbles of fetid gas from the bottom, and its floating islands of dead dogs, was a most potent agent in the induction of yellow fever. The men who were employed to clear it out were almost stifled with its effluvia, and the inhabitants of its shores died in great numbers of the fever. The cellars that sent up such a deadly gas from the pools of water which filled them as to kill the very flies hovering over them, were recognized by Dr. Drysdale as efficient causes of the disease."† Dr. Barton calls the meteorological and terrene conditions "the two blades in the shears of fate." But it will be observed that most of the meteorological conditions have a geographical or terrene relation to latitude and elevation, to moisture, atmospheric stagnation, miasm, etc., to locality and surroundings. The influence of climate—a vague expression so often used—is causative of the fever under consideration mainly, if not entirely, through the presence of noxious emanations and the hygrometric state of the atmosphere, which emanations and humidities have a direct dependence upon topographical circumstances, the nature of the soil, relative position to the sun, prevailing winds, weight of the air, and other causes.

I have repeatedly referred to dampness of site, of soil and surroundings, as a causative condition ever present where yellow fever is produced. Humidity, as an atmospheric condition, is deserving of a moment's further consideration. Frequent showers are not the only condition that gives rise to atmospheric humidity. High temperature, a large evaporating surface, a low site, and atmospheric quiescence, are conditions which may load the air heavily with moisture.

Atmospheric humidity acts in three ways in producing disease. First,

---

* Harper's Magazine, vol. xiii. p. 786.

† Harper's Magazine, vol. xv. p. 199.

it is indispensable to those putrid emanations which alter the healthy conditions of the air, and the greater the humidity the greater is the amount of miasm which the air is capable of holding in suspension, and, consequently, the more rapidly the infection. Second, by embarrassing cutaneous and pulmonary exhalation, moisture in the air causes secretions to be returned back into the fluids of the system and there act as poisons, disturbing the harmony of the functions not less effectually than though they were received into the system from without. According to Dalton, a man, under ordinary circumstances, exhales from the skin and lungs about thirty-seven ounces of fluid in twenty-four hours. These exhalations are imbued with effete matters from the organism, the retention of which must engender disease. Collard de Martegna has shown that the air expired contains $\frac{3}{1000}$ of purtrescent organic matters, any diminution in the exhalation of which must poison the system in the same manner and as effectually as though miasms were directly inhaled with the atmosphere. Third, the cutaneous exhalation of moisture and its evaporation from the surface acts directly as a cooling process, thereby rendering endurable a high temperature. In a damp atmosphere the cutaneous exhalation is not only less abundant, but its evaporation is retarded. Every one knows how much more oppressive is a hot and damp than a hot and dry atmosphere. A shower on a very hot day may actually cool the atmosphere several degrees, yet every one knows that the sun's heat is often even more oppressive than before. The reason of this is, the rain falling upon the hot and dry earth is rapidly converted into vapor, increasing the atmospheric humidity, and decreasing the cutaneous evaporation. A damp and retentive soil, marshes, rivers, lakes, and the sea-coast, are all sources of atmospheric humidity, and a cloudy sky, by checking radiation and retaining the heated vapors, may intensify it. Mountains may act in the same way by shutting out the cool and dry air, which is so healthful on the hills, and hemming in the hot air of the valley, filled with humidity and miasms. Dr. Lallemant tells us that when the yellow fever raged so deadly at Rio Janeiro, clouds often covered mountain tops; lightnings flashed, thunders rolled, and rains fell there and upon the plains beyond; but in the valley "the city and its vicinity were in the greatest apparent tranquillity of nature, the tranquillity of a cemetery." From what was said upon the geographical relations and terrene conditions of yellow fever, it will be seen that most of the conditions favorable to the production of great atmospheric humidity were present in all places where the disease prevailed epidemically.

Perhaps I have said more upon the subjects of putrid exhalations and humid atmosphere than seems necessary, but I have said less than I desired, for in the furtherance of this report, I shall have occasion to make frequent reference to those conditions, as causative of disease. Akin to the hygrometric state of the atmosphere is a high dew point. The dew point, I need not say, is the precise degree of temperature at which moisture is deposited from the atmosphere upon any cool substance. The higher the dew point then, the higher the temperature at which moisture is deposited, the greater the amount of watery vapor which the atmosphere is capable of holding in suspension. Dr. Barton places much stress upon a high dew point, and says yellow fever cannot prevail without it. He places the dew point of the fever at from 70° to 80°, and says it rarely exists when it is less than 60°. All that has been said in regard to atmospheric humidity will apply to this subject, and hence it is unnecessary to make additional remarks in regard to it. When noticing the plague I neglected to mention a high dew point as one of the usual conditions attending its manifestations. The dew point of the plague is high; but observations upon this point have been too limited to determine it exactly: it is probably between 60° and 70°.

Like the plague, yellow fever never appears above a certain elevation. What that certain elevation is, remains yet an unsettled question. Major Tulloch says it never has been known, in any climate, above 2500 feet above the sea. In Mexico, Humboldt says an elevation of 3243 feet forms the upper limit of range of yellow fever. It is highly probable that the fever ceases to appear at points far below these figures; certain is it that in this country, be the conditions of localities what they may, the disease has never yet appeared at an elevation above 400 feet. Memphis is the highest point in the United States where yellow fever has ever occurred; in fact, the disease has never appeared there but once, and then in 1828, when the fever prevailed as a mortal epidemic. Memphis is 400 feet above the Gulf of Mexico. When the disease prevailed in Rio Janeiro, an elevation of 400 feet upon the hills in the rear was found to present an insurmountable barrier. The reason of this exemption upon the hills, or at any considerable elevation, is, first and primarily, because of more thorough ventilation and less concentration of heat, the temperature is not high enough to sufficiently elaborate those causative conditions ever present where the fever exists, putrid exhalations and atmospheric humidity. The diminution of atmospheric pressure and the greater elasticity, and purity, and mobility of the air are other conditions favorable to exemption from the fever, which are usually present in elevated situations.

Is yellow fever contagious? This is a question that may be appropriately asked here. I do not propose to examine it at length, however inviting such a labor might be, but as briefly as possible make a statement of my convictions—convictions by no means hastily arrived at.

It would seem, from this hasty and imperfect sketch of yellow fever, as if a sufficiency of local causes had been found, in every instance, to account for the development of the disease, without invoking the aid of contagion and importation. This question is one upon which the profession is at present divided, and it is certainly one of paramount importance. If yellow fever is contagious, then sanitary cordons and quarantine regulations are the only safeguard of a city having commercial relations with a locality in which the disease prevails. If the disease be contagious, then it is presumption to allow those exposed to its influence to flee where they list, spreading the elements of contagion broadcast over the land. But if the disease be not contagious, then is it cruelty in the extreme to deny food and shelter to the fugitives fleeing from the mournful scenes of death; yea, it is base inhumanity to drive them back, disheartened and half starved to the afflicted city, to become themselves victims of the epidemic. Such cruelties and inhumanities have been acted over and over again, not only in dark ages and unenlightened countries, but in this country, even during the last epidemic. Of the cowardly selfishness enacted at Norfolk and Portsmouth, one writer says: "The ties of blood were sundered, bonds of alliance were as if they had never been, friend shuddered and shrank from friend, the sick and dying lay in hopeless despair, with none to moisten their parched lips, nor administer a soothing draught, while burial for the dead was with difficulty obtained." Men, women, and children fled by thousands to the pure air and healthy regions of the country, many not on their own account simply, but with the hope of saving a beloved wife, or dear little ones, from the ruthless ravages of the deadly visitant. In regard to these fugitives, another writer says: "In many instances a refuge was denied the unhappy fugitives, they were driven out from the places whither they had fled, and all intercourse with them was prohibited." Many were denied the comforts of life, and a home in the habitations of men, and were compelled to take shelter in barns, sheds, and school-houses, or to return and take their chance with the sick and dying that remained. If the disease be not contagious, that fact should be distinctly stated by the profession, and rightly understood by the public.

Much of the controversy that has originated upon the subject of contagion has been from a diverse conception or understanding of the term, and conflicting opinions might, in many instances, have been harmonized, had disputants rightly understood each other. It will be sufficiently accurate to answer my present purpose, to say that *all contagious diseases are produced by a specific poison, which poison is formed in the living fluids of the body; and they may be reproduced indefinitely, always with the characteristic symptoms of the disease, by a direct and unchanged*

*emanation from the body of a person under the active influence of such specific poison.* Diseases strictly contagious—originating in a specific virus—abundantly reproduce in the living fluids, and emit with the exhalations from the skin and lungs the same specific virus; which poison, when introduced into the blood of a healthy person, whether by inhalation or otherwise, is capable of producing in that person the same disease, and in like manner, the disease may be propagated indefinitely. Dr. Wood says: "There can be little doubt that the cause of yellow fever is peculiar and specific, as much so as that of small-pox or scarlatina."* From the explanation given above, it will be seen that the specific virus of contagious diseases, small-pox, for instance, originates *within* the living blood; the poison producing yellow fever may be specific, but all pathologists agree that it originates *external* to the body.

The contagious nature of yellow fever has been urged from the fact that the disease nearly always occurs first in a city near wharves, where vessels arrive from abroad and unload. To me this fact seems equally strong in favor of the non-contagious nature of the disease, for it is in such localities that those atmospheric and terrene conditions, previously referred to, are to be found in greater concentration and potency.

Again, it has been urged in disproof of the local origin of the disease, that the temperature and dew point are as high, and the atmospheric humidity and miasmatic accumulations are as great in Constantinople, for instance, where yellow fever never occurs, as in Philadelphia or Baltimore, at which places the disease has repeatedly prevailed as an epidemic.

Strong as this fact may at first appear, no stronger is needed to prove the non-contagiousness of the fever; for, if the disease be really contagious, it would be as likely to be imported into one maritime city as another; the quarantine regulations of Athens, and Rome, and Constantinople are probably not superior to those of New Orleans, and Vera Cruz, and Rio Janeiro. And even supposing they were, this argument of the contagionists is really worthless, for though all the conditions necessary for the production of yellow fever seem to be present, yet it is by no means certain that the peculiar and necessary arrangement of those conditions exists. Liebig has demonstrated that animal and vegetable matters, in a state of decomposition, yield different products according to varying meteorological, or, sometimes, unknown conditions.

Starch, gum-arabic, and cane-sugar are composed of precisely the same elements, and the two latter in precisely the same proportions, but, from a different arrangement of those elements, their sensible properties are quite dissimilar. It is highly probable that from some meteorological or

* Practice of Medicine, vol. i. p. 311.

unknown cause the products of animal and vegetable decomposition are different at Constantinople and at New Orleans, giving rise, in the former place, to a miasm, deficient in some condition or element requisite for the production of yellow fever. If this be not so, it may be supposed that the causative conditions requisite for the production of the fever, though all present, have not that peculiar arrangement necessary for the production of the disease under consideration. Under these circumstances, these causes will not prove inoperative, but will develop the plague, cholera, or typhus, whichever the conditions are exactly calculated to promote.

It has been claimed, in the third place, that individuals, going from an infected neighborhood into a healthy one, have become centres of a new infection; but this claim is, doubtless, without foundation. So far as I know, no well-authenticated case has ever occurred where the disease has apparently spread from one person to another, *in decidedly healthy localities*. It is true, that a person fleeing from a yellow fever locality, with all the seeds of the disease in his system, may himself have the fever in any locality, however healthy, but it is, probably, not true that he can impart the disease to others. Persons leaving New Orleans or Philadelphia, during the height of a yellow fever epidemic, may take the disease, and die with it at Columbus, Ohio, or Springfield, but they never can, in such localities, become the centres of a new infection. In fact, experience has demonstrated over and over again, that no fear of contagious communication is to be apprehended from the effects of emigration. These facts should be generally understood and appreciated, then may we look for a display of an enlarged humanity in the rural districts, near affected localities—then may we look for an abandonment of those theoretical notions of contagion, whose cruel fruits have, time after time, added thousands to the dark catalogue of death, by refusing a resting-place, shelter, and food to fugitives; compelling them to go back to deserted homes but to increase the demand for coffins and the labors of the sexton.

Dismissing the subject of contagion, I would say that yellow fever *cannot exist without the presence of a miasm, having a certain origin, and developed under certain conditions.* This origin, it is presumed, is partially animal and partially vegetable, with an active preponderance of the former. The fact that the disease only originates where men congregate, as in large cities, where the air is poisoned with human exhalations and the putrescent effluvia arising from decomposing human and animal excrements, and where, from circumstances, the very water is poisoned with percolations from privies and many other sources of animal putrescence, abundantly establishes the partial animal origin of the miasm. The lowness of site, the alluvial, argillaceous and undrained soil, the surrounding swamps, and, in some instances, the luxuriant growth and large deposit of sea-weed—conditions, with, perhaps, the exception of the last, always present, go to show the partial vegetable origin of this poisonous element of disease. The active preponderance of the animal origin derives support from the well-known fact that at New Orleans, and all other cities where the disease prevails, it first makes its appearance where the sources of animal filth are most abundant, and subsequently the attacks, in other streets, are in the order of the intensity of the miasmatic and other causes. In New Orleans, the animal and vegetable miasms have distinct centres; the animal miasmatic centre is along that side of the city facing the river, while the vegetable miasmatic centre is at the marshes bounding the opposite side. It is unnecessary to say more in regard to the origin of the causative miasm; the conditions requisite for its development are prolonged high temperature and dew point, with atmospheric humidity and stagnation, to intensify the miasmatic action. It is not difficult to see how miasms may disturb the healthy economy and produce disease. We all know that small-pox and glanders are produced by a specific poison, but that poison is animal matter in a state of decay. Some of us may know something experimentally of the painful and dangerous consequences resulting from wounds received in dissection; yet such consequences are produced by the reception into the system of a small portion of decaying animal matter. It has been demonstrated that man passes through his lungs more than 330,000 cubic inches of air daily; now, if this air contains miasms in the same minute proportion that it contains carbonic acid ($\frac{1}{2500}$), then more than twenty grains of poisonous exhalations might be received into the system through the lungs daily. This calculation is independent of what may be absorbed through the skin, or received with the food and drink. Some of you may remember the one hundred and forty-six English prisoners, confined for a night in the Black Hole of Calcutta; they struggled and pleaded vainly for fresh air, which was denied them; the morning light revealed only twenty-three ghastly survivors of the unhappy band!

It is needless to protract remarks to show the facility with which disease is developed, by the introduction into the system of animal or vegetable poisons, of which the atmosphere may become the vehicle.

If yellow fever is caused by a miasm, having a certain origin, and developed under certain conditions, it may be asked why it does not prevail annually at places where such miasm is supposed to be developed in the heat of every summer? At New Orleans, Vera Cruz, and Rio Janeiro, heat and moisture are annual visitants, and the filth of the cities, and the exhalations of the adjacent marshes, poison the air each year with their noxious gases. Why then is not yellow fever as regular in its outbreaks, in these cities, as are intermittent fevers among the suburbans adjacent to the swamps? This is a question, perhaps, not easy of solution; it has certainly staggered many, who else would have adopted the idea of the local origin of the disease. My opinion is that this question has its origin in, and is prompted by, an error in fact. The error consists in supposing that all miasms are alike; that the apparently similar conditions of heat, moisture, animal and vegetable decomposition, must develop the same products, and produce the same disease. I have already attempted to show that such is not the fact. Many circumstances cause the products of decomposition to vary, as, for instance, a diversity of decomposing substances, varying meteorological conditions, and dissimilar excitants to decomposition, decaying plants,—each yield an aroma peculiar to itself, of which peculiarity the sense of smell is abundantly cognizant. Emanations from marshes and graveyards are dissimilar; the effluvia from damp cellars and filthy stables are unlike; the gases arising from privies and from slaughter-houses or tanyards are not identical in sensible properties, and, probably, not in physiological effect. Thus dissimilar sources give dissimilar results.

The juice of grapes, decomposing under one condition, produces wine; under another, vinegar. A peculiar excitant added to a solution of cane-sugar converts it into alcohol and carbonic acid. Take two atoms of small-pox virus, inoculate a man with one atom and a cow with the other; the one will produce in the man a genuine small-pox, the other in the cow will produce a disease at least identical in nature. Take again two atoms of virus, one from a pustule on the man and the other from a pustule on the cow, introduce each into different men; and the one from the pustule on the man will produce again the genuine small-pox, the other atom will only produce a very insignificant disease, though allied to small-pox in nature, and identical in results. Thus a slight circumstance or varying condition may disarm a virus of its disease-producing intensity.

The elements of disease in a city, as New Orleans, for instance, may seem to be uniform, one year with another; the same sources of miasm exist within the city; the same marshes and lakes without; and the same sun influencing the whole. But these elements of decay, of exhalation, of miasm, must develop a poison of varying powers—a virus of different disease-producing qualities. This is the reason why these disease-producing elements give rise at one time to one disease, at another time to another and dissimilar one. Thus intermittent, remittent, and malignant bilious remittent fevers, yellow fever, cholera, erysipelas, diarrhœa, and dysentery may each, at different times, be developed, the which being determined by the peculiar products, or combination of products, of the disease-producing elements.

This varying manifestation of disease some have referred to a peculiar epidemic influence or poison. But the epidemic constitution, about which so much is said, it seems to me is nothing more nor less than *a peculiar miasm, developed under peculiar terrene and meteorological conditions.*

There is one idea, to which allusion has not been made, that is worthy of a moment's attention, which is, that *local miasms may be modified by miasms brought from abroad.* Thus heat and moisture, city filth and marsh miasms, may be operative with accustomed intensity, at Philadelphia, for instance, and yet yellow fever does not prevail. At this time a steamer may arrive from Rio Janeiro with no fever on board; may unload its cargo, containing perhaps many elements of putrescence; the offensive bilge-water is pumped out, and the vessel thoroughly cleansed. These new elements of putrefaction may act as an excitant or ferment upon the local miasms, producing new elements, all sufficient for the production of yellow fever. Some may suppose that this amounts to nothing less than contagion and importation. But a moment's reflection will show the fallacy of such an idea. The imported miasm is wholly insufficient to produce the disease itself, and it is only by a combination with local miasms, as a new excitant, that yellow fever is developed.

Perhaps I have protracted my remarks upon yellow fever beyond the limits of your patience. I have endeavored to show that this disease can only exist in the presence of certain atmospheric and terrene conditions; that the terrene conditions at least are under the control of man, and that the atmospheric are alone inadequate to its development.

A yellow fever epidemic is but the voice of an inexorable law, silently proclaiming man's sinful negligence of the laws of health and of life. When man will do his duty to himself and to his fellow-man, "when the putrid, decaying, noisome atmosphere exhaled by churchyards, slaughter-houses, the tanks of dirty water companies, cess-pools, sewers, crowded dwellings, etc., is purified and dissipated," then will epidemic poisons no longer remain in undisturbed possession of the earth and the air.

It is not to be presumed that certain portions of the earth's surface were destined to be devastated and depopulated by periodical visitations of deadly epidemics. The doctrine of Talfourd is, that "the immense regions of unbounded fertility, long succession of spicy groves, trackless pastures, watered by ocean rivers, formed to let in wealth to great continents, and islands which lie calmly on the breast of crystal seas, were not created for eternal solitude and silence." The doctrine of Talfourd is doubtless correct, and if man will do his duty, in removing the terrene conditions of disease, the time will come when yellow fever epidemics will cease to depopulate the tropical and sunny regions of the earth.

The author would remark that the preceding paper was written three years ago, and for a year past has been in the hands of the editors of the REVIEW, waiting its turn for publication. This explanation seems the more necessary, as he has made no use of, or reference to, the proceedings of the *Sanitary Association*, which has convened and published its transactions in the mean time. Those transactions have not fallen under his observation, and consequently could not have influenced his opinions, or materially altered the foregoing, had it just come from his pen.

